# Immune Complexes Impaired Glomerular Endothelial Cell Functions in Lupus Nephritis

**DOI:** 10.3390/ijms20215281

**Published:** 2019-10-24

**Authors:** Linlin Wang, Helen Ka Wai Law

**Affiliations:** Department of Health Technology and Informatics, Faculty of Health and Social Science, The Hong Kong Polytechnic University, Hung Hom, Hong Kong, China; linlin.wang@connect.polyu.hk

**Keywords:** immune complexes, glomerular endothelial cells functions, lupus nephritis

## Abstract

Lupus nephritis (LN) is one of the most common and severe complications of lupus. However, the mechanisms for renal damage have not been well elucidated. There are evidences show that glomerular endothelial cells (GECs) are damaged in LN. Immune complexes can deposit in subendothelial area and could affect GEC functions. In the present study, we used heat-aggregated gamma globulin (HAGG) to simulate immune complexes and investigated their effects on GEC functions. Our results revealed that HAGG impaired different aspect of the GEC functions. HAGG changed cell morphology, upregulated the expression of active caspase-3, inhibited angiogenesis, and increased NO production in GECs. These results provide new clues for the mechanisms of renal damage and the pathology of LN.

## 1. Introduction

Lupus nephritis (LN) is one of the most common and severe complications of lupus. Renal involvement is a leading predictor of poor prognosis in lupus patients [1]. Numerous factors (genetic, environmental, infectious, hormonal, and immune) contribute to this complex pathogenesis [2,3]. However, the precise mechanisms for renal impairment are not thoroughly understood. The glomerular filtration membrane (GFM) is the structural and functional foundation of the kidney, consisting of glomerular endothelial cells (GECs), podocytes, and glomerular basement membrane [4]. Impairment in any component of the GFM can injure renal functions. Compared to podocytes, the role of GECs in LN has not been well elucidated. 

There are many clues indicating that endothelial cells are impaired in LN [5,6]. Importantly, renal vascular lesions were observed in 81.8% of LN patients and lesions with vascular immune complex deposit was the most common form [7]. Loss of endothelial cells in glomerular capillaries was observed in renal biopsies from crescentic LN patients, associated with the formation of necrotizing and cellular crescentic lesions, and contributing to the development of glomerular sclerosis and collapse [8]. Moreover, circulating endothelial cells, which were detached mature endothelial cells responding to microvascular injuries, were increased in lupus patients with renal symptoms than patients without [9]. In lupus-prone mice models, increased expressions of endothelial adhesion molecules in glomeruli, including E-selectin and P-selectin, were detected and showed associations with severity of glomerular lesions [10]. Besides, normal endothelial nitric oxide synthase (eNOS) function is involved in LN development. LN mice that lacked eNOS developed more severe diseases, with more glomerular crescentic and necrotic lesions, elevated inflammatory infiltrates and vasculitis, and decreased survival [11]. Transcriptional network analysis offered additional support for the importance of endothelial function in LN. Berthier and his colleague [12] used the transcriptional network approach to define the similarities and differences among human LN and three murine lupus models (NZB/W, NZM2410, and NZW/BXSB strains) in molecular terms and finally found 20 commonly shared network nodes, which reflected the key pathologic processes in LN. Among the 20 key nodes, four nodes reflected injured endothelial cell functions, including adhesion (VCAM-1), fibrinolysis (ANXA2), coagulation (F2R, or thrombin receptor RAP1), and decreased angiogenesis (VEGF-A).

Endothelial cells line the innermost surfaces of vessels and actively participate in multitudes of physiological processes [13]. When challenged by stresses, endothelial cells become activated and dysfunctional [14]. In LN, immune complexes are central players for disease pathogenesis and can be deposited in subendothelial, subepithelial, and mesangial areas [15,16,17]. Deposited immune complexes served as a stress and could affect GEC functions. Sun and colleagues [18] reported that immune complexes upregulated ICAM-1 and VCAM-1 expressions on human umbilical vein endothelial cells (HUVECs), and increased secretions of interleukin (IL)-6, IL-8, tumor necrosis factor (TNF)-α, and monocyte chemoattractant protein (MCP)-1. Our previous study used artificially synthesized heat-aggregated gamma globulin (HAGG) to simulate the immune complexes and showed that immune complexes suppressed autophagy in GECs [19]. Thus, in the present study, we further investigated on a series of GEC functions under the stimulation of HAGG in vitro and found that cell morphology, cell death, angiogenesis, and intracellular nitric oxide production were impaired, which shed lights on the mechanisms of renal damage and the pathology of LN. 

## 2. Results

### 2.1. HAGG-induced GEC Morphology Changes

Cell morphology is a large-scale synthetic result of global precisely-regulated biological processes of cells, controlled by the interactions among cytoskeletons, membrane, membrane-bound proteins, and the extracellular environment. Changed cell morphology reflects changed cell physiology and functions. Cell morphology is commonly used as a measurement of the outcomes of various stimulations [20]. HAGG was prepared as an artificial substitute of immune complexes. When heated, disulfide bonds were formed among monomeric IgGs, and large aggregated proteins were generated [21,22]. These heat-induced large covalent aggregates display multitude similarities with natural immune complexes in structures and biological behaviors. Thus, in our experiments, we used HAGG as a surrogate for immune complexes to stimulate GECs.

In our experiments, GECs were cultured in four conditions: complete medium (control), HAGG (400 μg/mL), TNF-α (10 ng/mL), or TNF-α plus HAGG, respectively. After eight hours of incubation, phase-contrast images were taken and randomly-reselected single cells were outlined. Cell morphology parameters, including cell area (unit: pixel^2^), perimeter (unit: pixel), circularity, and aspect ratio were measured and calculated by ImageJ software.

**Figure 1:**
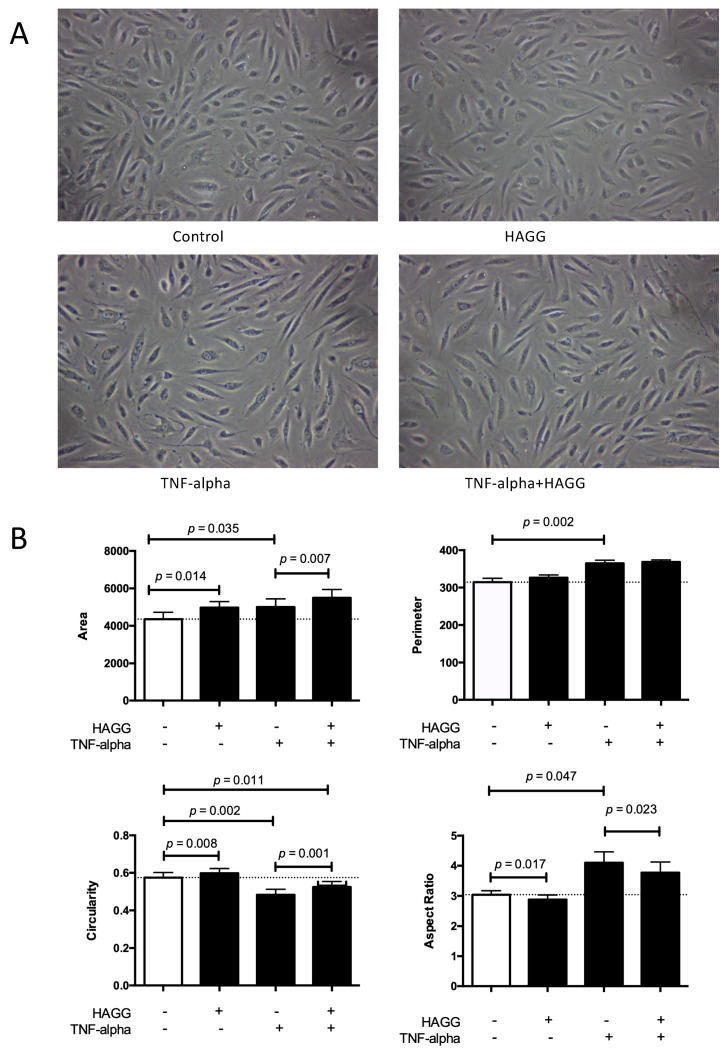
Cell morphology was changed after heat-aggregated gamma globulin (HAGG) treatment. Glomerular endothelial cells (GECs) were treated with complete medium (control), HAGG (400 μg/mL), TNF-α (10 ng/mL), or TNF-α plus HAGG, respectively, for 8 h. (**A**) Representative photo micrographs of cells after 8-h incubation with different stimuli. Magnification: 100 times. (**B**) Statistical analysis of cell morphology parameters. Cell area, perimeter, circularity, and aspect ratio, were qualified by ImageJ software. Dotted lines indicated the mean values of the variables under control conditions. Data was presented as mean ± SEM (*n* = 3). Paired *t*-tests were used to compare the groups under two different stimulations.

As shown in Figure 1, the HAGG treatment led to a significant increase in cell area (4979 pixel^2^ vs. 4358 pixel^2^, *p* = 0.014) and no significant difference in cell perimeter, in comparison with the control cells in complete medium. In geometry, the circle shape encloses the largest area for a given arc length (for plane curves). Therefore, these results indicate a rounder cell shape induced by HAGG. The significantly increased circularity (0.60 vs. 0.57, *p* = 0.008) and decreased aspect ratio (2.88 vs. 3.04, *p* = 0.017) also supported the same conclusion. TNF-α led to larger and more elongated GECs. Cells treated with TNF-α exhibited larger cell area (5001 pixel^2^ vs. 4358 pixel^2^, *p* = 0.035) and perimeter (364.7 pixel vs. 314.5 pixel, *p* = 0.002), lower circularity (0.48 vs. 0.57, *p* = 0.002), and higher aspect ratio (4.10 vs. 3.04, *p* = 0.047), compared with the control cells. Cells treated with TNF-α plus HAGG displayed a rounder shape than cells treated with TNF-α alone, demonstrated by the increased cell area (5495 pixel^2^ vs. 5001 pixel^2^, *p* = 0.007) under the same perimeter, increased circularity (0.52 vs. 0.48, *p* = 0.001), and decreased aspect ratio (3.77 vs. 4.10, *p* = 0.023).

In Figure 1A, we also show that cell densities were different after eight hours of incubation with different stimulations, although the initial seeding cell numbers were consistent. This observation prompted us to investigate cell viability and deaths under different stimulations.

### 2.2. HAGG Did Not Induce Necrosis in GECs

**Figure 2:**
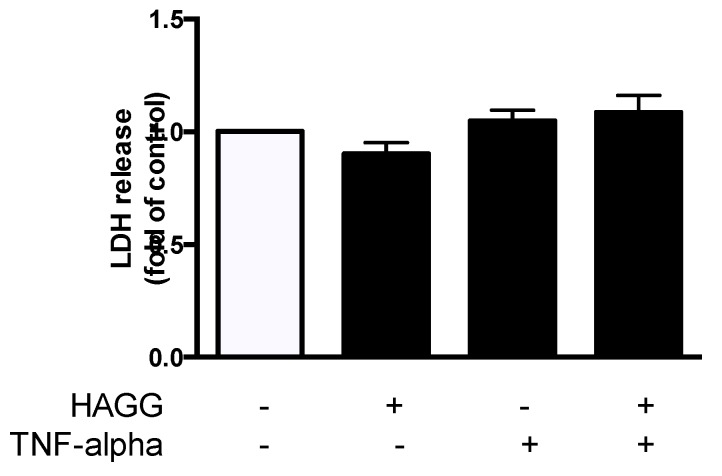
Effects of HAGG on GEC necrosis. GECs were treated with complete medium (control), HAGG (400 μg/mL), TNF-α (10 ng/mL), or TNF-α plus HAGG, for 48 h. Necrosis was measured using LDH release assay. Data was presented as mean ± SEM (*n* = 8) and analyzed by one-way ANOVA.

Our previous study revealed that GEC viability was decreased after HAGG stimulation [19]. We further measured cell necrosis by lactate dehydrogenase (LDH) release assay. GECs were cultured with complete medium, or treated with HAGG (400 μg/mL), TNF-α (10 ng/mL), or TNF-α plus HAGG, respectively, for 48 h. The released LDH activity in culture medium was measured. Results showed that HAGG treatment for 48 h did not increase LDH release (Figure 2). TNF-α nor TNF-α plus HAGG did not alter the LDH release.

### 2.3. HAGG Upregulated Intracellular Level of Active Caspase 3 in GECs

**Figure 3:**
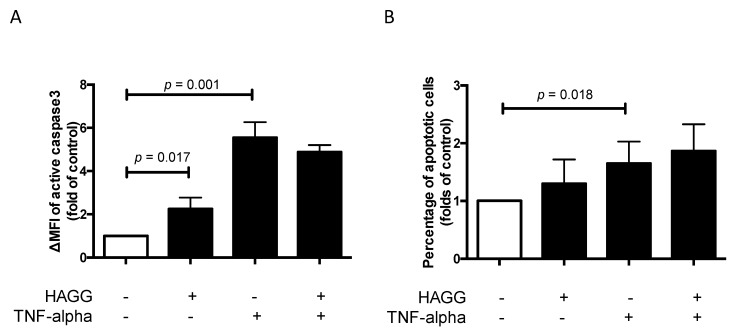
Effects of HAGG on GECs’ apoptosis. GECs were treated with complete medium (control), HAGG (400 μg/mL), TNF-α (10 ng/mL), or TNF-α plus HAGG for 48 h. (**A**) ΔMFI of active caspase 3 in GECs (*n* = 4). (**B**) Percentages of apoptotic cells measured by YO-PRP-1/PI assay (*n* = 6). Comparisons between two groups were analyzed by paired *t*-tests.

Next, apoptosis was measured by YO-PRO-1/PI assay and Active Caspase 3 assay using flow cytometry. In our experiment, HAGG treatment for 48 h resulted in an increased intracellular level of active caspase 3 (represented by increased ΔMFI of active caspase 3, 2.25 fold of control, *p* = 0.017, Figure 3A). The mean percentage of apoptotic cells (YO-PRO-1 positive and PI negative subsets) increased (1.30 fold of control, Figure 3B), although the increase did not show significant difference between HAGG-treated GECs and the control cells.

TNF-α is a typical inducer of apoptosis in endothelial cells by binding with its receptors and is often used as a positive control. TNF-α (10 ng/mL) treatment for 48 h led to increased ΔMFI of active caspase 3 (5.55 fold of control, *p* = 0.001) and percentage of apoptotic cells (1.65 fold of control, *p* = 0.018). There is no significant difference between the effects of TNF-α alone and TNF-α plus HAGG.

### 2.4. HAGG Suppressed GEC Tube Formation

A variety of endothelial cells, endothelial progenitor cells, and transformed endothelial cells have demonstrated the formation of tube-like structures rapidly in vitro when seeded on top of a reconstituted basement membrane extracellular matrix, such as Matrigel [23,24]. The formation of tube-like structures on basement membrane is specific to endothelial cells [25], and simulates multiple steps in the angiogenesis process, including endothelial cell adhesion, degradation of basement membrane, endothelial cell proliferation, migration, alignment, and tube formation. Therefore, this tube formation assay is widely used for assessing the angiogenesis properties of endothelial cells. 

**Figure 4:**
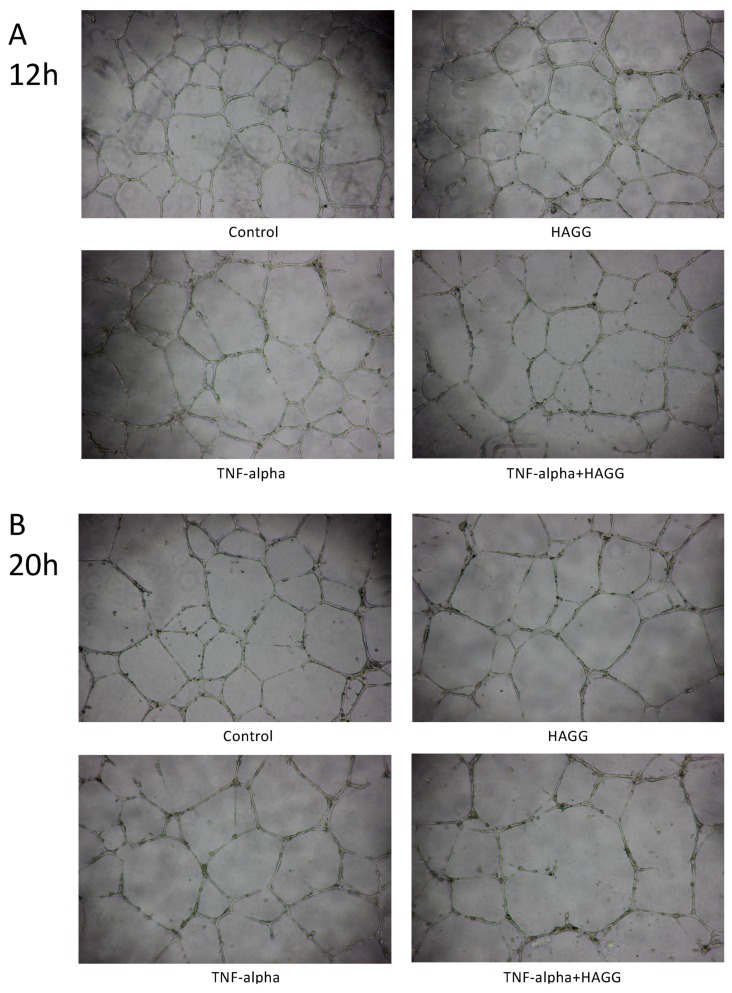

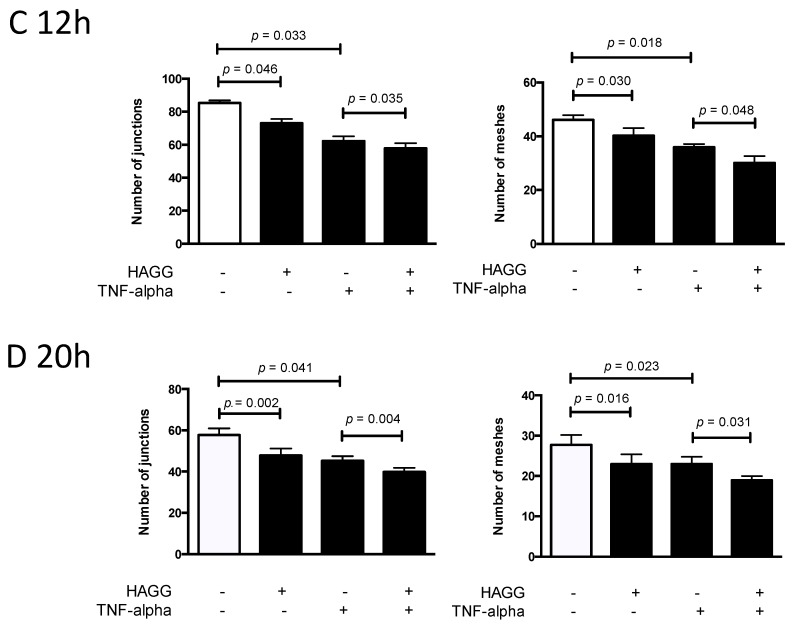
HAGG suppressed GEC tube formation on Matrigel. GECs were seeded on growth factor-reduced Matrigel and cultured with complete medium (control), HAGG (400 μg/mL), TNF-α (10 ng/mL), or TNF-α plus HAGG, respectively. Representative images of tube formation assay were captured after incubation for 12 h (**A**) and 20 h (**B**), using an inverted phase contrast microscope (magnification: 40 times). Quantifications of tube formation assays at 12 h (**C**) and 20 h (**D**), including the number of junctions and number of meshes, were evaluated by ImageJ software and plotted in column diagrams. Data were presented as mean ± SEM (*n* = 3). Comparisons between two groups were analyzed by paired *t*-test.

In our results, tube-like structures began to form within three hours after seeding GECs on the growth factor-reduced Matrigel-coated multi-well plates. Two parameters (the number of junctions and the number of meshes) were used to describe the complexity of the tube-like structures [26,27]. As shown in Figure 4A,C, after 12 h of incubation, HAGG-treated GECs formed less junctions (73 vs. 85, *p* = 0.046) and less meshes (40 vs. 46, *p* = 0.030) than control cells, suggesting suppressed tube formation ability. TNF-α also inhibited tube formation, with a decreased number of junctions (62 vs. 85, *p* = 0.033) and meshes (36 vs. 46, *p* = 0.018), when compared with the control cells. Combined stimulation with TNF-α and HAGG further suppressed tube formation, when compared with the effects of TNF-α alone (number of junctions: 58 vs. 62, *p* = 0.035; number of meshes: 30 vs. 36, *p* = 0.048). 

For tube formation assay observed at 20 h, the network structure became sparse, with less meshes (control: 46 vs. 28, *p* = 0.003) and less junctions (control: 85 vs. 58, *p* = 0.007, Figure 4C,D). Results at 20 h revealed a similar response to the individual stimulation, as shown at 12 h (Figure 4B,D). In sum, HAGG treatment for 20 h suppressed GEC tube formation (number of junctions: 48 vs. 58, *p* = 0.002; number of meshes: 23 vs. 28, *p* = 0.016; compared with control cells). GEC tube formations were also suppressed by TNF-α (number of junctions: 45, *p* = 0.041; number of meshes: 23, *p* = 0.023, compared with control cells). Additional HAGG with TNF-α led to further suppressions on tube formations (TNF-α plus HAGG vs. TNF-α: number of junctions: 40 vs. 45, *p* = 0.004; number of meshes: 19 vs. 23, *p* = 0.031 (Figure 4B,D).

### 2.5. HAGG Alleviated the Intracellular Nitric Oxide Production in GECs Stimulated by TNF-α

Nitric oxide (NO) is a membrane-permeable signaling molecule, which is mainly synthesized by NOS. NO is involved in a variety of biological processes and is essential for endothelial cell functions and vascular homeostasis, including regulation of blood tone, inhibition of plate aggregation and leukocyte adhesion, and suppression of cell proliferation [28,29,30]. However, overproduction of NO exhibits deleterious effects. NO can inhibit cytochrome C oxidase, leading to ATP depletion. NO also belongs to reactive nitrogen intermediates. NO and its relevant productions, peroxynitrite (ONOO-) and N_2_O_3_, can nitrosylate, nitrate, and oxidize proteins, DNA, and lipids, resulting in cytotoxicity [31,32]. Thus, in this section, intracellular NO production in GECs was measured using a NO-specific probe 4-amino-5-methylamino-2,7-difluorofluorescein diacetate (DAF-FM-DA). 

**Figure 5:**
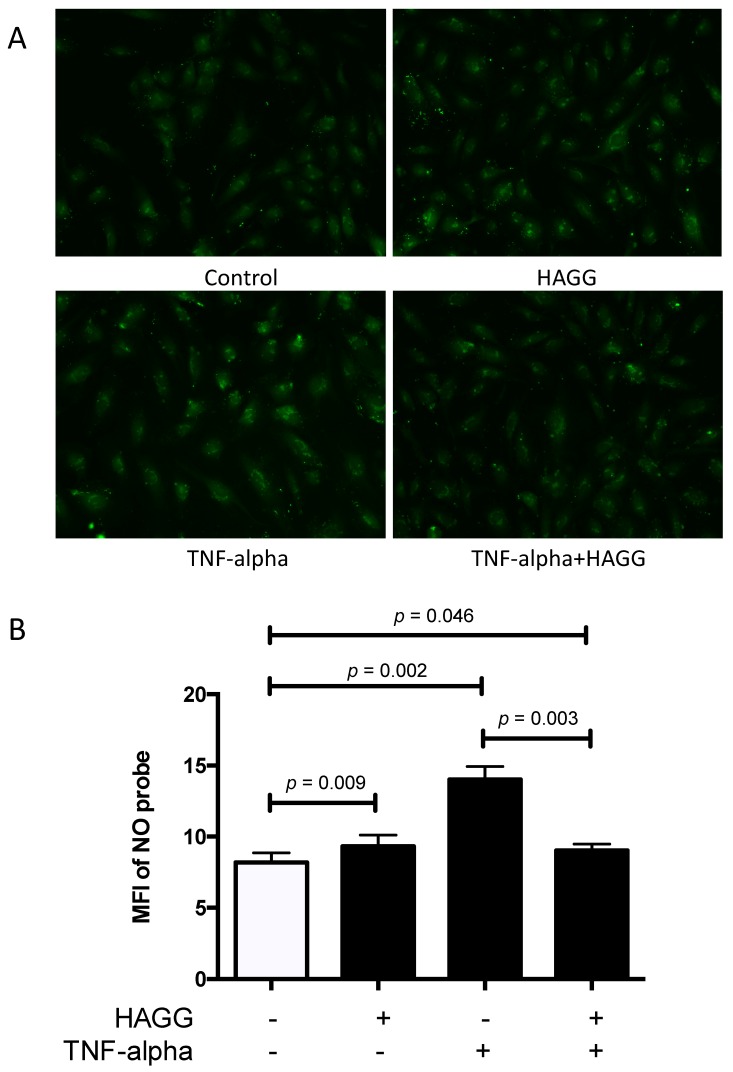
HAGG induced intracellular nitric oxide (NO) production in GECs. GECs were cultured with complete medium (Control), HAGG (400 μg/mL), TNF-α (10 ng/mL), or TNF-α plus HAGG, for 24 h. (**A**) Representative fluorescent images of GECs stained with DAF-FM probe, indicating intracellular NO production (magnification: 200 times). (**B**) Statistical analysis of the mean fluorescence intensity (MFI) of DAF-FM probe per cells. Data were presented as mean ± SEM (*n* = 4). Comparisons between two groups were analyzed by paired *t*-test.

In our experiments, GECs were incubated with complete medium, or treated with HAGG (400 μg/mL), TNF-α (10 ng/mL), or TNF-α plus HAGG, respectively, for 24 h. After staining with DAF-FM-DA probe for 20 min, images were captured by the camera attached to a fluorescence microscope and the mean fluorescence intensity (MFI) of DAF-FM probe per cell was measured by ImageJ software. As summarized in Figure 5, incubation with HAGG for 24 h slightly increased the intracellular NO production (MFI of NO probe was 1.12-fold of the control, *p* = 0.009). TNF-α alone led to significant increase in NO production (the MFI of the NO probe was 1.68-fold of control, *p* = 0.002). However, the combination of TNF-α and HAGG did not have a synergistic effect. Rather, the NO production induced by TNF-α was alleviated by HAGG (MFI of NO probe in TNF-α+HAGG vs. TNF-α: 9.02 vs. 14.01, *p* = 0.003). The NO production stimulated by TNF-α+HAGG was similar to that of HAGG alone (1.10 fold of control, *p* = 0.046). 

## 3. Discussion

Endothelial cells are metabolically active, rapidly responsive, and versatile cells, which are crucial in multitudes of physiological and pathological processes. There are various evidences indicating impaired endothelial cell functions in LN. GECs, especially, are important resident parenchymal cells in the kidney and participate in the formation of glomerular filtration barrier, which is the structural and functional foundation for the kidney. GEC function evaluations are important for understanding LN pathogenesis and clinical diagnosis. Thus, in this study, we incubated GECs with HAGG and inflammatory cytokines, and analyzed cell morphology, necrosis, apoptosis, tube formation, and intracellular NO production, which reflected several important aspects of endothelial cell functions. 

### 3.1. Cell Morphology and Cell Function

Normal cell morphology is fundamental for cell functions. Changed cell morphology reflects in changed cell functions. Our results revealed that GECs displayed a rounder cell shape after HAGG treatment, demonstrated by increased cell area, unchanged perimeter, increased circularity, and decreased aspect ratio. GECs became larger and elongated after TNF-α stimulation. Combination of TNF-α and HAGG resulted in a rounder shape than GECs treated with TNF-α alone. 

There are published papers investigating the relationships among cell morphology, biomechanics, cytoskeletal dynamics, and other cell functions. Stroka and colleagues observed that HUVECs became larger and elongated after TNF-α treatment for eight hours, supported by an increased cell area and aspect ratio, which was consistent with our results. Meanwhile, TNF-α-treated extended endothelial cells displayed increased contraction forces (measured by traction-force microscopy), and reduced migration speed, corresponding to the increased aspect ratio. Endothelial cells became softer after TNF-α treatment [33]. Szczygiel et al. also stimulated human dermal microvascular endothelial cells with TNF-α for 1–24 h. Changed cell shapes—from spherical to longitudinal—were observed. Meanwhile, longer stimulation with TNF-α (more than 6 h) resulted in progressive decrease in cell stiffness, F-actin depolymerization, and increased NO production [34]. Roca-Cusachs and colleagues reported that cell elongation decreased cell stiffness (measured by atomic force microscopy) [35]. These results remind us that the rounder cells caused by HAGG incubation in our experiments may indicate increased stiffness. Cell attachment, migration, even NO production properties, may be affected by changed morphology and cytoskeleton arrangement.

### 3.2. Angiogenesis

Angiogenesis is commonly defined as a process of generating new blood vessels from the pre-existing vasculature. Angiogenesis contains multiple steps, including endothelial cell degradation of the local basement membrane, cell migration toward stimulus (sprouting), cell proliferation, and cell reorganization for tubular structure and lumen formation [36]. Tube formation assay simulates most of these steps in angiogenesis and thus is widely used to assess endothelial cell angiogenesis [24].

Our results revealed that both HAGG and TNF-α suppressed GEC tube formation on Matrigel. The combination of HAGG and TNF-α further inhibited GEC tube formation. There were evidences supporting the inhibitory effects of TNF-α on endothelial cell tube formation. Hsu et al. reported that TNF-α inhibited HUVEC migration and capillary tube formation [37]. Du et al. also reported that TNF-α suppressed tube formation and induced cell apoptosis of endothelial progenitor cells, which were endothelial precursors and crucial for angiogenesis and neovascularization [38]. However, the effects of TNF-α on angiogenesis are controversial. There are also reports indicating that TNF-α promoted angiogenesis [39,40]. Moreover, Sainson and colleagues reported that continuous stimulation of TNF-α inhibited angiogenesis, while a pulse stimulation of TNF-α followed by normal medium culture promoted angiogenesis [41]. Their results implicated that not only the type of cytokines, but also the duration of stimulation, affects angiogenesis. For acute inflammation in vivo, TNF-α is cleared rapidly, which resembles pulse stimulation. Whereas in chronic inflammatory diseases and SLE, TNF-α is persistent in tissues and may cause suppressed angiogenesis.

Angiogenesis is precisely regulated [42]. Among numerous factors, VEGFs and their receptors, angiopoietins and their Tie2 receptors are key regulators for angiogenesis [43,44]. Some clinical observations implicate suppressed angiogenesis in LN. Messenger RNA expression of VEGF in the kidney biopsy samples from proliferative LN patients (Class III or IV) was lower than that from the control samples. Reduced immunohistochemistry staining of VEGF was also observed in glomeruli from LN patients [45]. Cross-species transcriptional network analysis revealed that decreased VEGF expression in kidney samples was commonly shared in three murine LN models and human LN patients [12]. Transcriptional analysis of glomeruli isolated by laser-capture microscopy revealed that gene expression of VEGF was decreased in renal biopsies from LN patients, in comparison with the control samples [46]. Wongpiyabovorn et al. also reported that a SNP (+405 GG) at the exon 1 in *VEGF* gene was associated with LN patients with low VEGF mRNA expression and with LN with end-stage renal disease [47]. Besides, serum concentration of the anti-angiogenic Ang-2 was increased in LN patients compared with control subjects, and positively correlated with SLEDAI scores [48,49]. Our results showed that HAGG suppressed GEC tube formation, which may provide another explanation for the abnormal angiogenesis in LN.

### 3.3. Intracellular NO Production

NO is a soluble and versatile molecule. Physical concentration of NO is essential for endothelial functions. However, overproduction of NO exhibits cytotoxic effects. There were evidences indicating that NO production was increased in LN and that this increased NO production might be damaging. Weinberg and colleagues reported elevated NO production in LN mice by measuring urinary excretion of nitrite/nitrate (in mice receiving nitrate-free diet). Disease manifestations were alleviated by NOS inhibitors [50]. Enhanced NO production was also reported in lupus patients (using serum nitrite and citrulline as surrogate markers), correlated with disease activities [51]. Belmont and colleagues reported similar results that NO production was increased in vascular endothelium from lupus patients [52]. Our results revealed that HAGG alone slightly increased NO production in GECs, which echoed the data from the above-mentioned studies. However, HAGG added to TNF-α ameliorated the stimulatory effect of TNF-α in NO production. This paradoxical effect of HAGG needs to be explored and further investigations are needed to decipher the intriguing relationship between different pathways.

### 3.4. Cell Viability and Cell Death

Loss of endothelial cells in glomerular capillaries was observed in renal biopsies from patients with lupus nephritis [3]. In our previous study, cell viability was measured by the CCK-8 method and the results suggested that HAGG induced slightly decreased cell viabilities in GECs [19]. In this study, the results reveal that necrosis was not induced by HAGG according to LDH release assay. For apoptosis, HAGG upregulated intracellular level of active caspase 3 in GECs, but no difference in the percentage of apoptotic cells was observed. Normally, decreased cell viabilities mainly result from two reasons: cell death (including necrosis and apoptosis) and cell growth inhibition. Next, we will investigate the effects of immune complexes on glomerular endothelial cell cycle and proliferation.

## 4. Materials and Methods

### 4.1. Cell Culture

Primary human GECs were purchased (ACBRI 128, Cell Systems, Kirkland, WA, USA) and cultured as previously described [19]. Briefly, human GECs were cultured in CSC complete medium (containing 10% serum) activated with CultureBoost (Cell Systems), at 37 °C in a humidified atmosphere of 5% CO_2_-95% air. Culture surfaces were pre-coated with Attachment Factor (Cell Systems), and cells were seeded at a density of 1 × 10^5^ cells/mL. Cells at passage 5-11 were used in this project. 

### 4.2. Heat-Aggregated Gamma Globulin Preparation

HAGG was artificially synthesized to simulate immune complexes. Purified human monomeric IgG (10 mg/mL, Sigma-Aldrich Corporation, St. Louis, MO, USA) was heated at 62 °C for 30 min, as described [53], and diluted in phosphate buffer saline (PBS) to the desired concentration for the experiments.

### 4.3. Cell Morphological Analysis

GECs were seeded in 6-well plates, at a density of 0.3 million cells per well. When the cells reached 70–80% confluency, stimulations were added. After 8 h, the cells were observed and photographed using an inverted phase contrast microscope (Eclipse TS100, Nikon, Tokyo, Japan), at 100 times magnification. Cell morphology was assessed by multiply quantitative measurements, all of which were performed by ImageJ software. Outlines of individual GECs were traced manually using Polygon selection tool. Cell area, perimeter, circularity, and aspect ratio, were measured. Perimeter is the length of the outside boundary of the selection. Circularity was defined as $4\pi \times \frac{\left[Area\right]}{{\left[Perimeter\right]}^{2}}$, with a value ranging from 0 to 1. The value of 1 indicates a perfect circle, while when the value approaches 0, it indicates an increasingly elongated shape. Aspect Ratio was defined as $\frac{\left[Major\;Axis\right]}{\left[Minor\;Axis\right]}$, which can also reflect the degree of cell elongation. At least 30 cells were randomly chosen under each condition. The results were from three independent experiments.

### 4.4. Apoptosis Assay by Flow Cytometry

Apoptosis was measured using Dead Cell Apoptosis Kit with YO-PRO-1 and PI (Invitrogen, Thermo Fisher Scientific, Inc., Eugene, OR, USA) and PE Active Caspase 3 Apoptosis Kit (BD Biosciences, San Diego, CA, USA), according to the manufacturers’ instructions using flow cytometry (BD FACS Aria III, BD Biosciences). 

YO-PRO-1/PI assay: After incubation with different stimuli, GECs were harvested, washed and re-suspended in cold PBS at a concentration of 1 million cells per 1 mL PBS. Each 1 mL cell suspension was incubated with 1 μL YO-PRO-1 stock solution and 0.5 μL propidium iodide (PI) stock solution for 20 min on ice. Then cells were distinguished and analyzed by flow cytometry. Cells without staining and stained with single dye were used to perform standard compensation. Live cells are defined by the double negative population, while the dead cells are defined by the double positive population. Apoptotic cells are YO-PRO-1+PI-. Representative plots are included in Figure S1.

Active caspase 3 assay: Harvested GECs were re-suspended and fixed in BD Cytofix/Cytoperm solution (1 million cells/0.5 mL) for 20 min on ice. The cells were then washed twice with BD Perm/Wash solution at a volume of 0.5 mL buffer/1 million cells at room temperature and incubated with the active caspase 3 antibody solution for 30 min at room temperature. Eventually, the cells were washed and re-suspended in 250 μL BD Perm/Wash solution and analyzed by flow cytometry.

### 4.5. Necrosis Measurement

Necrosis associated with different treatments in GECs was evaluated by measuring the released LDH in the cell medium, using the CytoTox 96 Non-radioactive Cytotoxicity Assay (Promega, Madison, WI, USA), following the modified protocols [54]. Two sets of replicates for each condition were used. Wells with medium alone without cells were used as blank control. At the end of the treatment period, 2 µL (2% of total volume) of Triton X-100 (Sigma-Aldrich Corporation) was added to one set of the wells to thoroughly degrade the cell membranes and release the total LDH. The plate was then centrifuged for 5 min at 1000 rpm. 50 µL of the supernatants from the two set of wells (with and without Triton X-100) were transferred to a new clean 96-well plate, and mixed with 50 µL CytoTox 96 Reagent. The CytoTox 96 Reagent was pre-prepared by mixing 12 mL room-temperature Assay Buffer to a bottle of Substrate Mix. Then the plate was incubated at room temperature for 20 min in the dark. The absorbance at 490 nm was measured by Benchmark Plus Microplate Reader (Bio-Rad, Hercules, CA, USA). 

### 4.6. Tube Formation Assay

The tube or vascular-like structure formation by endothelial cells was assessed on Matrigel Growth Factor-Reduced (Product #356231) Basement Membrane Matrix (Corning, Bedford, MA, USA), as previously described [55]. Briefly, the Matrigel was thawed overnight at 4 °C and 300 μL of Matrigel was added to each well of the 24-well plate. The plate was then incubated at 37 °C for 30 min, to ensure complete gelation of the matrix. GECs (30,000 cells per well) were seeded on top of the solidified Matrigel layer in 200 μL culture medium, with different stimulations, and incubated at 37 °C. Subsequently, the tube networks were observed using an inverted phase contrast microscope (Eclipse TS100, Nikon), at 40 times magnification, and recorded at 12 h and 20 h after seeding. Five randomly selected non-overlapping fields were photographed for each condition. Each experimental treatment condition was tested in triplicate. The images were analyzed using Angiogenesis Analyzer in ImageJ software and checked by manual counting. The degree of tube formation was quantified by measuring the number of junctions, the number of meshes, and total tube length. 

### 4.7. Intracellular Nitric Oxide Measurement

Intracellular NO production was detected by fluorescence microscopy using the NO-specific probe 4-amino-5-methylamino-2,7-difluorofluorescein diacetate (DAF-FM-DA, Thermo Fisher Scientific Inc.), according to the manufacturer’s instructions. GECs were grown on glass coverslips in 24-well plates (30,000 cells per well). After stimulations, the cells were washed with pre-warmed PBS (with Mg^2+^ and Ca^2+^) and incubated with diluted DAF-FM-DA (5 µM, in PBS with Mg^2+^ and Ca^2+^) for 20 min at 37 °C. The cells were then washed to remove excess probes and incubated in fresh CSC complete medium for 30 min, allowing complete de-esterification of the intercellular diacetates. The cells were then fixed in 4% paraformaldehyde and visualized using a fluorescence microscope (Eclipse 600, Nikon), equipped with a 495-nm excitation and 515-nm emission filter. Mean fluorescence intensity per cell was analyzed using ImageJ software.

### 4.8. Statistical Analysis

Results for morphology analysis, LDH release assay, tube formation assay, and NO production were presented as mean ± SEM. Other data were expressed as mean ± SD (indicated in the legends). Data were analyzed by Statistical Product and Service Solutions (SPSS) version 22 (IBM Corp, Armonk, NY, USA) and plotted by Prism 5.0 Software (GraphPad, San Diego, CA, USA). Normality tests were performed. Comparisons between two groups were examined by paired *t*-test. Comparisons among multiple groups were analyzed by one-way ANOVA with post hoc tests. *p* value < 0.05 was regarded as statistically significant. 

## 5. Conclusions

In this study, we incubated GECs with HAGG and examined their effects on endothelial cell functions. Our results revealed that HAGG changed cell morphology, upregulated the expression of active caspase-3, inhibited angiogenesis, and increased NO production in GECs. Although some damages may be mild, their effects on endothelial cells cannot be ignored, especially in chronic diseases such as LN. The effects of immune complexes on GEC functions provide new clues for the mechanisms of renal damage and the pathology of LN. Maintaining GEC functions may be a new target and method for LN therapy.

## Supplementary Materials

Supplementary materials can be found at https://www.mdpi.com/1422-0067/20/21/5281/s1.

## Abbreviations

eNOS	Endothelial nitric oxide synthase
GFM	Glomerular filtration membrane
GECs	Glomerular endothelial cells
HAGG	Heat-aggregated gamma globulin
HUVECs	Human umbilical vein endothelial cells
IL	Interleukin
LDH	Lactate dehydrogenase
LN	Lupus nephritis
MCP	Monocyte chemoattractant protein
MFI	Mean fluorescence intensity
NO	Nitric oxide
TNF	Tumor necrosis factor
