# Supplementary material for: Immune Complexes Impaired Glomerular Endothelial Cell Functions in Lupus Nephritis
Source: Int J Mol Sci. 2019 Oct 24;20(21):5281. doi: 10.3390/ijms20215281 (PMC6862593; doi:10.3390/ijms20215281)
Supplement: Supplementary file 1 [file ijms-20-05281-s001.pdf]

International Journal of *Molecular Sciences*

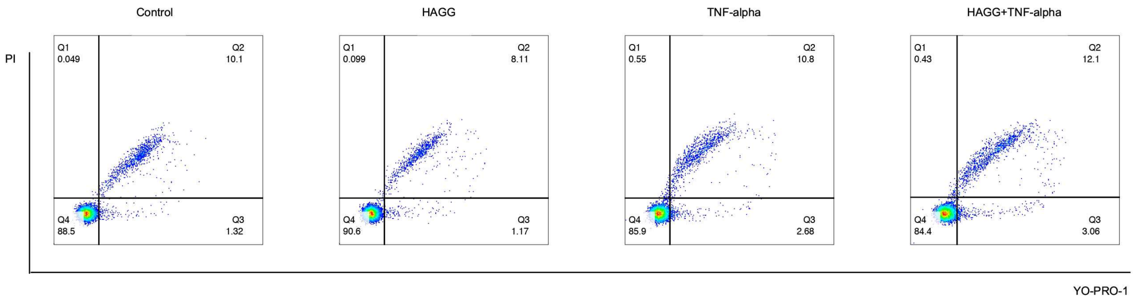

Figure S1. Representative flow cytometry plots for YO-PRO-1/PI assay. GECs GECs were treated with complete medium (control), HAGG (400 μg/mL), TNF-alpha (10 ng/mL), or TNF-alpha plus HAGG, for 48 hours. Live cells are defined by the double negative population, while the dead cells are defined by the double positive population. Apoptotic cells are YO-PRO-1+PI-. These are representative plots from one of the six independent experiments.
